# A new model for identification of HBV-related pre-ACLF

**DOI:** 10.1016/j.jhepr.2022.100554

**Published:** 2022-08-17

**Authors:** Rajiv Jalan, Vinay Sundaram

**Affiliations:** 1 Liver Failure Group, University College London Medical School, Royal Free Hospital Campus, Hampstead, London, UK; 2 European Foundation for the Study of Chronic Liver Failure, Barcelona, Spain; 3 Karsh Division of Gastroenterology and Comprehensive Transplant Center, Cedars-Sinai Medical Center, Los Angeles, CA, United States

*Rest but never quit. Even the sun has a sinking spell each evening. But it always rises next morning. At sunrise, every soul is born again. - Muhammed Ali*

Acute-on-chronic liver failure (ACLF) is a clinical condition that occurs in patients with cirrhosis and is characterised by organ failure(s) and high short-term mortality. The syndrome is clinically complex as it involves the cause of the underlying cirrhosis (**P**redisposition), an event that leads to the transition of the patient from a clinically stable situation to developing ACLF (**I**njury; precipitating event), body’s response to the injury (**I**nflammation-**I**mmune deficiency - **I**nfection) and one or more **O**rgan failures (liver, coagulation, kidney, brain, circulation, respiration).^1^ Despite this complexity, it is intriguing that the prognosis of the patients at the time of presentation to the hospital with an acute decompensating event can be made with a high degree of accuracy using a simple organ failure scoring system, referred to as the CLIF organ failure score.^2^

In the CANONIC study, the question of the role of the aetiology of cirrhosis, type and precipitating events, severity of inflammation and type and number of organ failures on the outcomes of patients was explored but not in any degree of detail.^3^ It is interesting to note that, in that study, the only factors that were considered to predict short-term mortality were the CLIF organ failure score, white cell count and age.^2^ Following the publication of that paper, it has become clear that determination of prognosis with more accuracy needs to be nuanced. For example, cirrhotic ACLF patients with different aetiologies have different outcomes, notably those with non-alcoholic fatty liver disease and primary biliary cholangitis.^4^^,^^5^ Also, not all organ failures are the same and the severity of organ failure needs to be considered in defining prognosis. For instance, patients with circulatory failure requiring large doses of inotropes and those with respiratory failure are likely to have high mortality even with liver transplantation.^6^^,^^7^

It is against this backdrop that one needs to try and better understand the role of precipitating events. First, one must consider why stable patients with underlying cirrhosis can develop such a rapid transition to multiorgan dysfunction. The exact mechanism is unclear, but it seems that due to many years of liver injury and consequent metabolic disturbances, bacterial translocation and low-grade systemic inflammation, the patient’s organs and immune system are primed to the effect of a second insult, which leads to manifestations of ACLF. Several pathways have been reported to be involved but the best studied involve the Toll-like receptor 4 pathway, activation of the inflammasome, metabolic disturbances (such as hyperammonemia), mitochondrial dysfunction, endotoxemia and senescence.^1^

The important paper by Wang, Tan, Wang, Zheng, and Huang *et al.* in the present issue of the *Journal* explores issues around the role of precipitating events in acutely decompensated patients with HBV-related cirrhosis who progress from no ACLF to ACLF.^8^ Data on 1,167 patients with acute decompensation (AD, no ACLF) and 197 patients with ACLF was derived from 2 large, prospective, multicentre studies in China. One of the central aims of the study was to identify risk factors for the occurrence of ACLF in patients with no ACLF at the time of presentation, a group referred to as pre-ACLF. Ninety-four patients with AD went on to develop ACLF and this group had a 90-day mortality that was like those with ACLF, an observation that is consistent with the PREDICT study that was performed in Europe.^9^ Analysis of clinical and pathophysiological variables showed that the occurrence of ACLF in this population was independently associated with 4-variables that related to precipitating events, namely, HBV reactivation, HBV flare, superimposed infection on HBV and bacterial infection; liver function parameters, international normalized ratio, and bilirubin; and a marker of systemic inflammation, neutrophil/lymphocyte ratio. Using these parameters, they developed a new score, which they validated in a second cohort of patients, showing a C-index of 0.862, which was better than the traditional prognostic models including the CLIF-AD and the model for end-stage liver disease score.

These data are very compelling and need to be put into context of the data available from the PREDICT study, which was specifically designed to better understand the role of precipitating events.^9^ This was a multicentre, prospective, observational study in Europe that focussed on patients with AD of cirrhosis. Most patients had underlying alcohol-related cirrhosis. It was remarkable that in 61% acutely decompensated patients with no ACLF, no specific precipitating illness was found. The commonest precipitating events that accounted for about 90% of the ‘known’ precipitating events were bacterial infections and alcoholic hepatitis. Interestingly, the presence of these precipitating events was significantly greater in the pre-ACLF patients who went on to develop ACLF. In addition, the greater the number of precipitants, the worse the clinical outcome. Data regarding the clinical outcomes and their relation to the number of precipitating events is not available in the Chinese cohort.^8^

Taken together, the study by Wang, Tan, Wang, Zheng, and Huang *et al.* brings to the literature an important, new perspective on the importance of the type of precipitating event, as well as introducing a new prognostic score to define the risk of future ACLF in patients with AD and no ACLF.^8^ Models such as this are an important unmet need as recognition that these patients are likely to develop ACLF would enable urgent, aggressive interventions to reduce the risk of progression to ACLF and consequent mortality. Furthermore, this patient population would be ideal to include in clinical trials of novel agents to allow for selection of patients at highest risk of progression to ACLF. However, the major limitation of this study is that it can only be applied to patients with HBV, which is limited to a few geographical areas of the world. Furthermore, the relatively low positive predictive value of only around 50% limits its clinical applicability to select patients for clinical trials. Finally, the requirement for specialised investigations for the diagnosis of some of the precipitating events would prevent correct prognostication at the time of hospitalisation.

A large effort is now being organised by several investigators around the world to identify biomarkers that may allow for accurate identification of patients with pre-ACLF. Inflammatory markers, characterisation of immune cell function, cell-death markers, measurement of matrix proteins and use of agnostic approaches such as metabolomics and whole blood transcriptomics are starting to produce interesting data that may refine clinical prognostic models, whilst providing novel therapeutic targets.^10^ Nevertheless, the data presented here provide the first clear indication of the importance of the type of precipitating event in defining progression of acutely decompensated patients with HBV-related cirrhosis from no ACLF to ACLF. These models should be further developed for other aetiologies such as alcohol-related cirrhosis.

## Financial support

This project has not received any funding.

## Authors’ contributions

RJ and VS developed the idea for the article, which was written by RJ. Due to the premature death of VS, he was not able to review the final version nor approve the manuscript.

## Conflict of interest

RJ has research collaborations with Yaqrit. RJ is the founder of Yaqrit Limited, which is developing UCL inventions for treatment of patients with cirrhosis. RJ is an inventor of ornithine phenylacetate, which was licensed by UCL to Mallinckrodt. He is also the inventor of Yaq-001, DIALIVE and Yaq-005, the patents for which have been licensed by his University into a UCL spinout company, Yaqrit Ltd. VS does not have any conflicts to declare.

Please refer to the accompanying ICMJE disclosure form for further details.
