# Supplementary material for: A new model for identification of HBV-related pre-ACLF
Source: JHEP Rep. 2022 Aug 17;4(10):100554. doi: 10.1016/j.jhepr.2022.100554 (PMC9472060; doi:10.1016/j.jhepr.2022.100554)
Supplement: Multimedia component 1 [file mmc1.pdf]

ICMJE DISCLOSURE FORM

Date: 220822
Your Name: Rajiv Jalan
Manuscript Title: A new model for identification of HBV-related pre-ACLF
Manuscript number (if known): JHEPR-D-22-00329

In the interest of transparency, we ask you to disclose all relationships/activities/interests listed below that are related to the content of your manuscript. "Related" means any relation with for-profit or not-for-profit third parties whose interests may be affected by the content of the manuscript. Disclosure represents a commitment to transparency and does not necessarily indicate a bias. If you are in doubt about whether to list a relationship/activity/interest, it is preferable that you do so.

The following questions apply to the author's relationships/activities/interests as they relate to the current manuscript only.

The author's relationships/activities/interests should be defined broadly. For example, if your manuscript pertains to the epidemiology of hypertension, you should declare all relationships with manufacturers of antihypertensive medication, even if that medication is not mentioned in the manuscript.

In item #1 below, report all support for the work reported in this manuscript without time limit. For all other items, the time frame for disclosure is the past 36 months.

		**Name all entities with whom you have this relationship or indicate none (add rows as needed)**	**Specifications/Comments (e.g., if payments were made to you or to your institution)**
		**Time frame: Since the initial planning of the work**	
1	All support for the present manuscript (e.g., funding, provision of study materials, medical writing, article processing charges, etc.) **No time limit for this item.**	____None	
		**Time frame: past 36 months**	
2	Grants or contracts from any entity (if not indicated in item #1 above).	____None	
3	Royalties or licenses	Mallinckrodt Pharma	My university licensed ornithine phenylacetate, a drug to treat encephalopathy. May receive future royalties
4	Consulting fees	____None	

5	Payment or honoraria for lectures, presentations, speakers bureaus, manuscript writing or educational events	Grifols	Lecture Fee
			Grant Panel Fee
6	Payment for expert testimony	____None	
7	Support for attending meetings and/or travel	____None	
8	Patents planned, issued or pending	____None	
9	Participation on a Data Safety Monitoring Board or Advisory Board	____None	
10	Leadership or fiduciary role in other board, society, committee or advocacy group, paid or unpaid	European Foundation for the Study of Chronic Liver Failure	Scientific Director
11	Stock or stock options	Yaqrit Ltd	Scientific Founder
		Cyberliver Ltd	Scientific Founder
		Hepyx Ltd	Scientific Founder
12	Receipt of equipment, materials, drugs, medical writing, gifts or other services	____None	
13	Other financial or non-financial interests	____None	

Please place an "X" next to the following statement to indicate your agreement:

X I certify that I have answered every question and have not altered the wording of any of the questions on this form.
